# Caffeine Affects Time to Exhaustion and Substrate Oxidation during Cycling at Maximal Lactate Steady State

**DOI:** 10.3390/nu7075219

**Published:** 2015-06-30

**Authors:** Rogério Santos de Oliveira Cruz, Rafael Alves de Aguiar, Tiago Turnes, Luiz Guilherme Antonacci Guglielmo, Ralph Beneke, Fabrizio Caputo

**Affiliations:** 1 Santa Catarina State University, Human Performance Research Group, Rua Pascoal Simone, 358, Coqueiros, Florianópolis, Santa Catarina 88080-350, Brazil; E-Mails: cruz.rso@gmail.com (R.S.O.C.); tiagoturnes89@gmail.com (T.T.); fabrizio.caputo@udesc.br (F.C.); 2 Federal University of Santa Catarina, Physical Effort Laboratory, Pantanal, Florianópolis, Santa Catarina 88040-900, Brazil; E-Mail: luizguilherme@cds.ufsc.br; 3 Abt. Medizin, Training und Gesundheit, Inst. Sportwissenschaft und Motologie, Philipps Universität Marburg, Marburg 35037, Germany; E-Mail: ralph.beneke@staff.uni-marburg.de

**Keywords:** fat metabolism, submaximal performance, muscle glycogen, endurance

## Abstract

This study analyzed the effects of caffeine intake on whole-body substrate metabolism and exercise tolerance during cycling by using a more individualized intensity for merging the subjects into homogeneous metabolic responses (the workload associated with the maximal lactate steady state—MLSS). MLSS was firstly determined in eight active males (25 ± 4 years, 176 ± 7 cm, 77 ± 11 kg) using from two to four constant-load tests of 30 min. On two following occasions, participants performed a test until exhaustion at the MLSS workload 1 h after taking either 6 mg/kg of body mass of caffeine or placebo (dextrose), in a randomized, double-blinded manner. Respiratory exchange ratio was calculated from gas exchange measurements. There was an improvement of 22.7% in time to exhaustion at MLSS workload following caffeine ingestion (95% confidence limits of ±10.3%, *p* = 0.002), which was accompanied by decrease in respiratory exchange ratio (*p* = 0.001). These results reinforce findings indicating that sparing of the endogenous carbohydrate stores could be one of the several physiological effects of caffeine during submaximal performance around 1 h.

## 1. Introduction

The ergogenic effects of caffeine on endurance performance are now well established [1,2]. A wide variety of mechanisms have been proposed to explain such effects in the human body, which ranges from increased reliance on fat metabolism [3,4,5], attenuation of the rate of muscle glycogenolysis [3,6], and alterations in central neurotransmitters or neuromuscular function [7,8]. However, measurements of substrate utilization by indirect calorimetry during whole-body exercises occasionally fails to support the theory of enhanced fat oxidation [1,2,9], leading to the notion that caffeine ingestion has minimal effects on the metabolism in working muscles [9].

In the majority of studies, a protocol of exercise tolerance at a given percentage of maximal oxygen uptake (V̇O_2_max) was used, which has been shown to have similar sensitivity to that of time-trials for changes in endurance [10]. However, despite being the most widely used exercise intensity index, a given percentage of V̇O_2_max is possibly not the best functional definition. This is because the parameters that discriminate between selected ranges or clusters of similar metabolic response characteristics (*i.e.*, lactate threshold, maximal lactate steady state (MLSS), and V̇O_2_max) have highly variable relationships among each other in different individuals [11,12]. In other words, assigning exercise intensities based on percentage of V̇O_2_max could actually lead participants to undergo distinct exercise intensity domains, yielding markedly different physiological strain characteristics. Thus, merging such responses from different participants into a single average could be misleading with respect to inferences about the effect of caffeine intake on metabolism.

On the other hand, the blood lactate response during exercise is recognized as a better predictor of endurance performance than V̇O_2_max [13]. Furthermore, the blood lactate response is a widely used tool for estimating relative exercise intensity and the metabolic responses at submaximal exercise intensities [14,15]. MLSS represents the highest intensity that can be performed in the absence of progressively increasing in blood lactate concentrations (BLC), which means that the oxidative energy metabolism accounts for the energy provision in active muscles [14]. Indeed, MLSS does not indicate only a given workload but rather an exercise intensity above which metabolism changes qualitatively; the transition from aerobic to partly anaerobic metabolism as indicated by continuing net lactate increase [16]. Additionally, exercise tolerance at MLSS has shown a large negative correlation with the percentage of the energy derived from carbohydrates [17]. This latter result is consistent with the notion that MLSS is highly dependent on carbohydrates metabolism [14,15]. Therefore, in comparison to a given exercise intensity related to V̇O_2_max, exercising to exhaustion at the intensity corresponding to MLSS appears as a more individualized strategy to assess effects of caffeine under a given metabolic situation during submaximal exercise.

Since carbohydrate combustion has proven decisive for exercise tolerance at MLSS workload, some sparing of the endogenous carbohydrate stores by an increased reliance on fat oxidation may help explain the ergogenic effects of caffeine intake on exercise tolerance. Thus, the aim of the present study was to analyze the effects of caffeine ingestion on physiological response and exercise tolerance during cycling at MLSS.

## 2. Experimental Section

This study was a randomized, double-blind, crossover trial with two arms: Placebo and caffeine. Participants completed five to seven laboratory test sessions on separate days within a 3-week period: An initial incremental test to determine the peak power and V̇O_2_max, two to four constant workload exercise bouts to determine MLSS, and two exercise bouts until exhaustion at MLSS in each condition (*i.e.*, caffeine or placebo). During the last two trials, a set of physiological and metabolic responses were compared between treatments at specific time points to evaluate the effects of caffeine intake on MLSS.

### 2.1. Subjects

Eight physically active male ranging in age from 20 to 31 years, with an average height of 176 cm (range: 169–193 cm) and an average body mass of 77 kg (range: 64–102 kg), volunteered and gave written informed consent to participate in this study, which was performed according to the declaration of Helsinki and approved by the Ethics and Research Committee of the Santa Catarina State University. All participants were apparently healthy nonsmokers who were taking no medication or nutritional supplementation. They were injury-free and practiced physical activity at least twice a week. Before each test, individuals were asked to abstain from intense exercise and products that contained caffeine for at least 48 h and to keep the same dietary intake profile for at least 72 h.

### 2.2. Procedures

All cycling tests were conducted on an electronically braked cycle-ergometer (Ergo 167 Cycle, Ergo-Fit, Pirmasens, Germany) with pedal frequency maintained at 70 rpm. The interval between each test was at least 48 h. The participants were instructed to arrive at the laboratory in a rested and fully hydrated state within 2–3 h postprandial, and to avoid strenuous exercise in the 48 h preceding a test session. Each participant was tested at the same time of day (±1 h) to minimize the effects of circadian variation. All cycle tests were performed in a temperature-controlled laboratory (21 ± 1 °C).

The incremental test started with a power of 1 Watt per kg of body mass and was increased by 0.5 Watt per kg of body mass every third minute until exhaustion. Participants were verbally encouraged to continue as long as possible. The test ended at the point of voluntary exhaustion or when participants were unable to maintain the predefined pedaling rate. During the test, participants breathed through a facemask, and pulmonary oxygen uptake was measured continuously (Quark PFT ergo, Cosmed, Rome, Italy). Data were averaged for each 15 s period. Maximal oxygen uptake and peak power were defined as the highest 15 s oxygen uptake value and the power output attained at exhaustion, respectively.

For MLSS determination, the power output set for the first test corresponded to 70% of the peak power measured during incremental test to minimize the number of tests per subject. Each constant workload test lasted 30 min and capillary blood samples (25 µL) were collected by micropuncture at the earlobe and then stored into microcentrifuge tubes containing 50 µL NaF (1%) at minutes 10 and 30 for the electrochemical determination of BLC, using the enzyme electrode technology (YSI 2300 STAT, Yellow Springs, OH, USA). If during the first test a steady state or decrease in BLC was observed, further subsequent 30 min constant work rate tests with a 10 Watts higher work rate were performed on separate days until no steady state could be maintained. Alternatively, if a clearly identifiable increase in BLC occurred or if the test could not be completed due to exhaustion, further constant work rate tests were conducted with reduced work rate (10 Watts). The MLSS workload was defined as the highest power output at which BLC did not increase by more than 1 mmol·L^−1^ between minutes 10 and 30 of the constant load test [18].

On the following two occasions, participants were then asked to perform a test until exhaustion at MLSS after the administration of either caffeine (6 mg per kg of body mass) or dextrose (placebo). The opaque capsules were administered with 250 mL of water in a randomized, double-blinded manner. To prevent foreknowledge of treatment assignment, the sequence order (caffeine-placebo or placebo-caffeine) was implemented using a sequentially numbered container (an allocation schedule by allocation concealment to prevent selection bias and to protect the assignment sequence until allocation). Allocation and assignment for each participant was performed by an independent, blinded staff member of our laboratory. Following supplementation, participants rested for 50 min after ingestion, and then completed a 5 min warm-up at 50% of peak power. After that, they were instructed to rest passively sitting on the bike. One hour after consuming the capsule, participants started the test. They were verbally encouraged to exercise as long as possible, and exhaustion was reached when the subject was unable to maintain the required pedaling rate (70 rpm). During these tests, capillary blood samples were collected at pretest, in the tenth and the thirtieth minutes of exercise, and at exhaustion to determine blood glucose and lactate concentrations. The oxygen uptake (V̇O_2_), carbon dioxide output (V̇CO_2_), respiratory exchange ratio (RER), minute ventilation (V̇E), and heart rate (HR) data were measured breath by breath, subsequently reduced to averages of 30 s and compared in the tenth and thirtieth minutes of exercise, and at exhaustion. Subjects were able to ingest water (ad libitum) throughout the time to exhaustion protocols, whereby the facemask was temporarily removed. Time to exhaustion protocols are among the most reliable of measures of endurance performance. The apparently poor reliability of time to exhaustion (coefficients of variation ≥ 10%) is an artifact of the relationship between exercise duration and power output. When duration-power relationship was used to convert changes in time to exhaustion into equivalent changes in power output in a constant-duration time trial, it was found that time to exhaustion is among the most reliable of measures of endurance performance [19].

### 2.3. Statistical Analyses

Comparisons were performed with the mixed linear modeling procedure of the IBM SPSS statistics (Version 19.0, IBM Corporation, New York, NY, USA), using the subject term as a random effect. Treatment was a fixed effect, and order of treatment was included as an additional fixed effect to account for continuing familiarization or other order effects. For cardiorespiratory and metabolic responses, moment was also included as a fixed factor. The changes in BLC from tenth to thirtieth minute of exercise were analyzed using the BLC at MLSS determination as a covariate. The uncertainties were expressed as 95% confidence limits and all tests were analyzed at an alpha level of 0.05.

## 3. Results

During the incremental test, participants achieved a peak mechanical power of 272 ± 37 W and presented a mean V̇O_2_max of 3.95 ± 0.61 L·min^−1^, or 51 ± 5 mL·kg^−1^·min^−1^ when expressed relative to body weight. The constant power at MLSS was 192 ± 27 W and corresponded to 73% ± 11% of V̇O_2_max.

Using the concentrations observed at MLSS determination as a control, caffeine ingestion affected the changes in BLC from tenth to thirtieth minute of exercise when compared to placebo. Caffeine condition resulted in an average increase of 1.4 mmol·L^−1^ (confidence limits of ±0.6 mmol·L^−1^, *p* < 0.001), while the increases in BLC after placebo ingestion were only 0.4 mmol·L^−1^ (confidence limits of ±0.6 mmol·L^−1^, *p* = 0.145). The typical error of BLC measurements was 0.4 mmol·L^−1^ (confidence limits of ±0.4 mmol·L^−1^).

**Figure 1:**
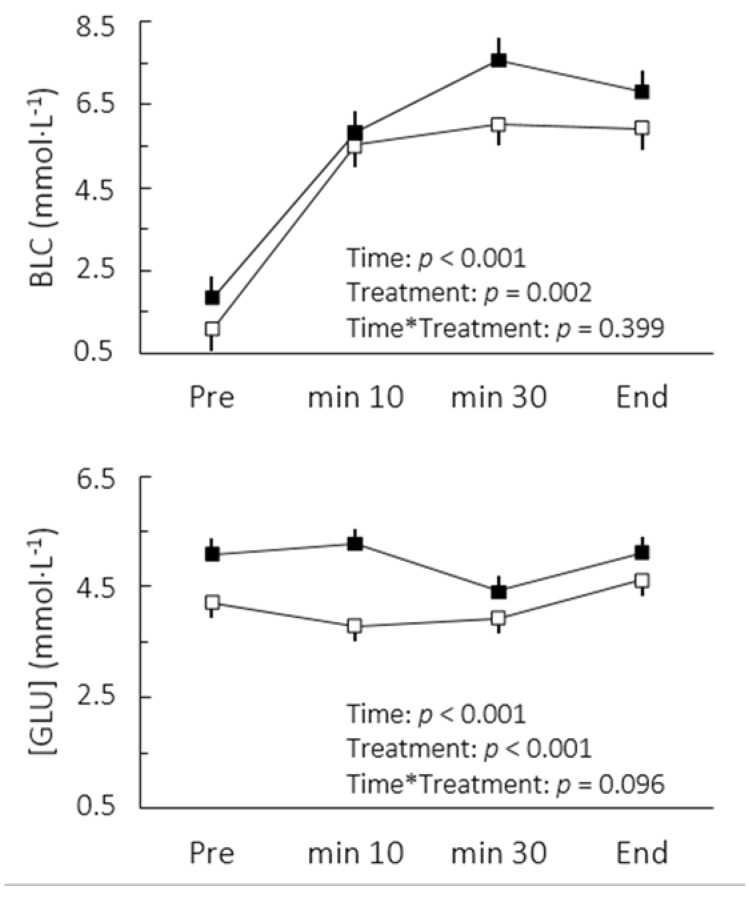
Blood lactate (BLC) and glucose ([GLU]) concentrations after caffeine (**▪**) and placebo (**▫**) ingestion during cycling to exhaustion at maximal lactate steady state. Values are mean ± SE.

**Figure 2:**
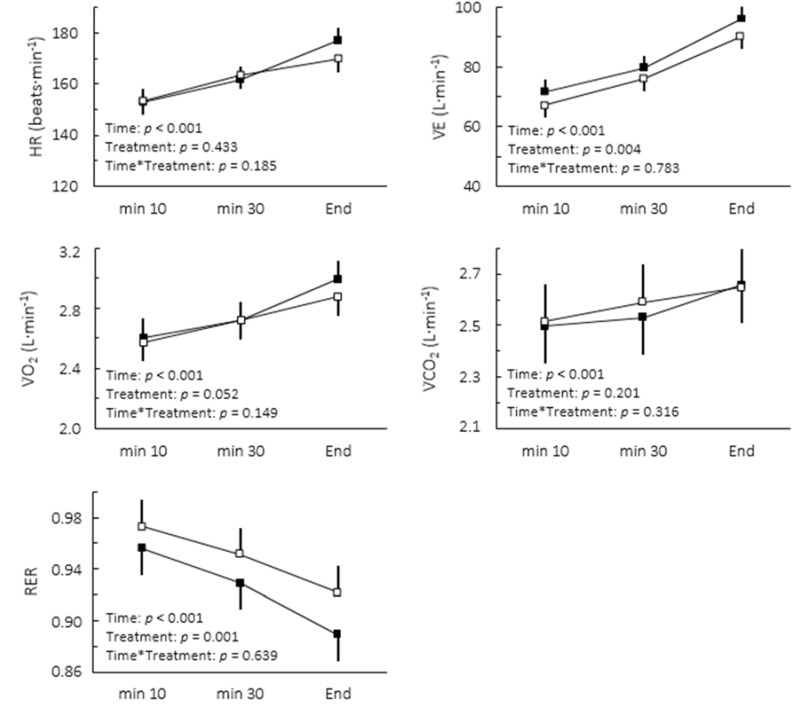
Cardiorespiratory responses to caffeine (**▪**) and placebo (**▫**) during cycling to exhaustion at maximal lactate steady state. Values are mean ± SE.

There was an improvement of 22.7% in time-to-exhaustion at MLSS workload following caffeine ingestion relative to placebo (Caffeine: 70.0 ± 4.1 min; Placebo: 57.0 ± 4.1 min, confidence limits of ±10.3%, *p* = 0.002). This change was accompanied by increases in blood lactate and glucose concentrations (Figure 1). The comparison of cardiorespiratory responses to exercise in both conditions is depicted in Figure 2. While no interaction between moment and treatment was found, caffeine intake significantly decreased the overall RER (2.8%, confidence limits of ±1.6%) and increased V̇E (5.5% confidence limits of 3.4%).

## 4. Discussion

The aim of this study was to analyze the effects of caffeine ingestion on physiological responses and exercise tolerance during cycling at MLSS. Caffeine exerted a positive effect on time to exhaustion at MLSS workload and this improvement was accompanied by a decreased RER during whole-body exercise. These results suggest that carbohydrate sparing caused by improved fat oxidation may be one of the several physiological effects of caffeine during submaximal exercise. The lower RER after caffeine ingestion also strengthen earlier findings showing the importance of the maintenance of endogenous carbohydrate stores during submaximal exercise [20,21], and particularly one of the factors affecting exercise tolerance at MLSS [15,17].

The test-retest variability in reliability studies of time to exhaustion is roughly 10% [10], which coincides with our own calculations of the typical error that was potentially overestimated by individual responses to caffeine [2,22]. Therefore, as the performance improvement (23%) was about twice the random variation from test to test, our results indicate that acute caffeine ingestion can evoke large enhancements in endurance at the MLSS workload for active males [23].

The increased sympathetic stimulation and/or a direct adenosine antagonism by caffeine has been shown and is thought to be responsible for increased adipose tissue lipolysis, which notably affect the time course of plasma free fatty acids concentration during exercise [9]. Indeed, the lower RER observed at MLSS suggests enhanced fat oxidation and depressed carbohydrate combustion after caffeine ingestion. Similar results were found by McNaughton *et al.* [24] during a 60 min time trial and by Ryu *et al.* [6] in cycling to exhaustion at 80% of V̇O_2_max preceded by 45 min at 60% of V̇O_2_max. In these studies, the performance improvements were based upon a greater reliance on fat metabolism, as indicated by increased free fatty acids concentrations and lower RER. In addition, it appears worthy to mention that some investigations reported elevated muscle citrate or cAMP concentrations with caffeine treatment during exercise at similar intensities [9,25].

However, some studies have failed to find any modification of the RER after caffeine ingestion (e.g., [1,2,9]). These conflicting results could be partially explained by the differences in the experimental design. In the present study, the cycling exercise was performed at an intensity characterized by a common physiological strain [26] instead of assigned at a given percentage of V̇O_2_max. Indeed, our active subjects showed a large range in the percentage of V̇O_2_max for MLSS intensity (56% to 88%) but the spread of the RER values between individuals was half of those reported in a study at which the exercise was assigned as 70% of V̇O_2_max [9]. If in the present study the intensity would have been fixed at 70% of V̇O_2_max for all participants, certainly they would have been exercising at very distinct exercise-intensity domains, resulting in markedly different physiological and metabolic responses between individuals [12]. If this is so, then there would be in many cases the presence of non-metabolic CO_2_ contributing for the miscalculations of fuel selection estimates.

Although we are aware that shifts in substrate metabolism are most unlikely to be the single aspect responsible for the caffeine-induced performance improvement during submaximal exercise [8,27,28], the present result is consistent with the negative correlation between the percentage of the energy derived from carbohydrates and exercise tolerance at MLSS workload reported by Billat *et al.* [17]. The reduced RER, probably reflecting a decrease in carbohydrate oxidation rate, means that the combined amount of muscle glycogen, lactate and blood glucose oxidized was reduced after caffeine ingestion. Even though blood glucose and lactate concentrations were increased in the caffeine trial throughout the experiment, evidence supporting a lower glucose or lactate uptake by the exercising leg is weak, with the decrease in clearance being attributed to inactive tissues [9]. On the other hand, earlier studies showed a decrease in muscle glycogen breakdown related to caffeine ingestion at submaximal intensities in both rats [6] and men [3], although not unanimously [9]. It is worthy to mention that decreases in muscle glycogen are thought to contribute to the reduction in force and cytosolic calcium concentration observed during fatiguing protocols [29], to play a protective role in the excitation-contraction coupling processes that is independent of its metabolic function [30], and to affect the central nervous system [31,32].

Although we accurately determined MLSS workload in this study, the BLC did not reach a steady state between the 10th and 30th minutes following caffeine ingestion. It could be argued at a first glance that caffeine actually lowered the mechanical power associated with MLSS. However, there appeared to be a delay for blood lactate stabilization rather than a disequilibrium per se (Figure 1). Indeed, an interrelationship between the BLC level and the BLC time constant has been reported [18]. At steady states between 6 and 8 mmol·L^−1^ as seen in the present study, a time constant between 7 and 11 min can be expected. Furthermore, it is important to note that the high reliability of the BLC found in MLSS tests administered without caffeine indicates a high test-retest reliability of MLSS, reinforcing our findings regarding higher lactate concentrations under delayed steady state conditions after caffeine uptake. In practical terms, the altered lactate response may affect MLSS determination. Thus, to ensure that the actual MLSS work rate is not underestimated, it seems advisable to avoid any dosage of caffeine during tests for MLSS determination, even though it is still unknown whether these effects would occur when doses of caffeine lower than 6 mg per kg of body mass are consumed.

The lack of a rigorous dietary control constitutes a limitation of the present study, as similar pre-exercise nutritional status is paramount to investigate the effects of caffeine intake on whole-body substrate metabolism. However, individuals were instructed to keep the same dietary intake profile for at least 72 h and to abstain from intense exercise for 48 h (both being confirmed by personal reports during each visit), which could be compromising similar pre-exercise levels of, for example, muscle glycogen. Based in the high reliability of the BLC response found during MLSS tests without caffeine, it seems unlikely that our subjects have not satisfactorily followed these instructions, since the BLC response at MLSS is highly sensitive to muscle glycogen levels [33]. Furthermore, the residual term of the mixed modeling procedure represents a measure of a within-subject random variation that could be increased by potential individual responses to caffeine ingestion [22]. For RER, this “inflated” value of typical error was 2.0% (95% factor limits of ×/÷1.3), suggesting very high reliability of RER measurements at MLSS. This value includes technological (e.g., calibration of equipment) as well as a biological variations, taking particularly into account the fact that subjects might not have strictly followed the instructions to replicate diet. Importantly, the rather low typical error (*i.e.*, ≤ 2%) permitted the discrimination of a 2.8% reduction on the overall RER after caffeine ingestion, through a satisfactory signal-to-noise ratio. Therefore, even if slight changes in baseline metabolic milieu between conditions had occurred, they seem to have not affected the finding of lower RER under caffeinated condition in the present study. Nevertheless, even though our results have proven robust to track the caffeine-induced changes in performance and substrate metabolism at MLSS, evidenced by tight confidence intervals, it would be interesting to replicate these results in larger samples, as a small sample may not always be representative of the target population. In addition, these results have to be confirmed in other populations, such as females and athletes.

## 5. Conclusions

In summary, caffeine exerted a positive effect on time to exhaustion at MLSS workload that was accompanied by shifts in substrate utilization during exercise. This strengthens the findings that sparing of the endogenous carbohydrate stores may be one of the several physiological effects of caffeine during submaximal exercise and perhaps one of the factors determining exercise tolerance at MLSS. We recommend future studies investigating how/whether caffeine ingestion affects MLSS workload.
